# Systemic IGF-1 gene delivery by rAAV9 improves spontaneous autoimmune peripheral polyneuropathy (SAPP)

**DOI:** 10.1038/s41598-018-23607-9

**Published:** 2018-04-03

**Authors:** Tong Gao, Nataliia Bogdanova, Sameera Ghauri, Gang Zhang, Jianxin Lin, Kazim Sheikh

**Affiliations:** Department of Neurology, McGovern Medical School at The University of Texas Health Science Center at Houston, Houston, TX 77030 USA

## Abstract

Spontaneous autoimmune peripheral polyneuropathy (SAPP) is a mouse model of chronic inflammatory demyelinating polyradiculoneuropathy (CIDP) in non-obese diabetic (NOD) mice null for costimulatory molecule, B7-2 gene (B7-2^−/−^). SAPP is a chronic progressive and multifocal inflammatory and demyelinating polyneuropathy of spontaneous onset with secondary axonal degeneration. Insulin-like growth factor 1(IGF-1) is a pleiotropic factor with neuroprotective, regenerative, and anti-inflammatory effects with extensive experience in its preclinical and clinical use. Systemic delivery of recombinant adeno-associated virus serotype 9 (rAAV9) provides robust and widespread gene transfer to central and peripheral nervous systems making it suitable for gene delivery in neurological diseases. A significant proportion of patients with inflammatory neuropathies like CIDP do not respond to current clinical therapies and there is a need for new treatments. In this study, we examined the efficacy IGF-1 gene therapy by systemic delivery with rAAV9 in SAPP model. The rAAV9 construct also contained a reporter gene to monitor the surrogate expression of IGF-1. We found significant improvement in neuropathic disease after systemic delivery of rAAV9/IGF-1 gene at presymptomatic and symptomatic stages of SAPP model. These findings support that IGF-1 treatment (including gene therapy) is a viable therapeutic option in immune neuropathies such as CIDP.

## Introduction

Neuropathic conditions grouped under chronic inflammatory demyelinating polyradiculoneuropathy (CIDP) are the commonest acquired chronic inflammatory neuropathies encountered clinically. The prevalence of CIDP in general population varies from 1.9–8.9 per 100,000^1–6^. These conditions are frequently characterized by inflammation, demyelination and secondary axonal injury, and potential responsiveness to immunomodulatory treatments. Extent and distribution of inflammation and axonal injury are important indicators of prognosis including fixed clinical deficits leading to substantial morbidity and disability^7^.

Typical CIDP is characterized by hematogenous leukocytes infiltration of the endoneurial compartment of peripheral nerves and/or nerve roots, resulting in axonal demyelination and/or degeneration. Perivascular mononuclear cells, predominantly monocytes/macrophages, and CD4/CD8 T lymphocytes infiltrate peripheral nervous system (PNS) and macrophage-mediated myelin stripping is the pathological hallmarks^8^. In addition to endoneurial inflammation, up-regulated plasma, serum or cerebrospinal (CSF) proinflammatory cytokine levels, including IL-17^9^ and IFNγ^10,11^, are also found to correlate with the acuity and severity of CIDP, suggesting that T cells play a critical role in the pathogenesis of CIDP including demyelinating and axonal nerve fiber injury.

Currently, corticosteroids (CS), intravenous immunoglobulin (IVIg), and plasmapheresis are used as first line, evidence based, immunomodulatory treatments for CIDP. Data from randomized controlled trials indicate that up to 2/3^rd^ of CIDP patients benefit from these treatments. However CS, IVIG, and plasmapheresis only provide short term benefits, many patients remain dependent on long-term treatment^12^. Moreover, a significant proportion of patients with CIDP do not or poorly responsive to current immunomodulatory therapies. Further, axonal loss tends to accumulate over time in patients with CIDP and current anti-inflammatory therapies do not have direct neuroprotective or proregenerative effects. In this context, development of new therapies with immunomodulatory and neuroprotective and/or regenerative properties is desirable.

Spontaneous autoimmune peripheral polyneuropathy (SAPP) is a reproducible mouse model of progressive inflammatory demyelinating neuropathy with secondary axonal loss in female nonobese diabetic (NOD) mice deficient in the costimulatory molecule, B7-2 (CD86). Generally, NOD B7-2^−/−^ mice develop inflammatory neuropathy in female animals starting at age 20 weeks, and 100% of females, 30% of males are affected by age 32 weeks^13^. The immunopathogenesis of SAPP has overlapping features with human CIDP. In SAPP model, endoneurial inflammation consists of CD4^+^/CD8^+^ T cells and monocytes/macrophages and adoptive transfer studies demonstrate that CD4^+^ T lymphocytes are central to the development of inflammatory neuropathy. Previous studies also demonstrate that proinflammatory cytokine IFNγ plays an obligatory role in the development of neuropathy as NOD B7-2^−/−^ and NOD.Aire^GW/+^ mice (a dominant G228W mutation of Aire gene) deficient in IFNγ are completely protected from disease^14,15^. Because of these overlapping immunopathogenic features, it is argued that SAPP is the most representative model of CIDP, and thus B7-2^−/−^ mice are used in our current studies examining the efficacy of insulin-like growth factor 1 (IGF-1) gene therapy.

IGF-1 is a pluripotent growth factor with multiple trophic functions in the peripheral nervous system. IGF-1 mostly uses IGF-1 receptor (IGF-1R) for its signaling, which belongs to tyrosine kinase receptor superfamily^16^. IGF-1R is expressed widely in all neural cells during development and throughout lifespan^17^. In peripheral nerve, Schwann cells also express IGF-1R^18^. For example, IGF-1/IGF-1R promotes neuronal survival, neurite formation and outgrowth in sensory, motor, and sympathetic neurons, and promotes Schwann cell survival, proliferation, differentiation, and myelination^19^. IGF/IGF-1R has also been reported to express in peripheral blood mononuclear cells (PBMCs)^20^. They modulate inflammation in a number of experimental paradigms. It is reported that IGF-1 can suppress proinflammatory Th1 responses, including IFN-γ production, and promote anti-inflammatory Th2 responses^21^. In this context, we examined the efficacy of IGF-1 in an animal model of CIDP due to its dual neurotrophic and immunomodulatory properties.

## Results

### Expression and distribution of IGF-1 following systemic administration of AAV9-IGF-1

**Figure 1:**
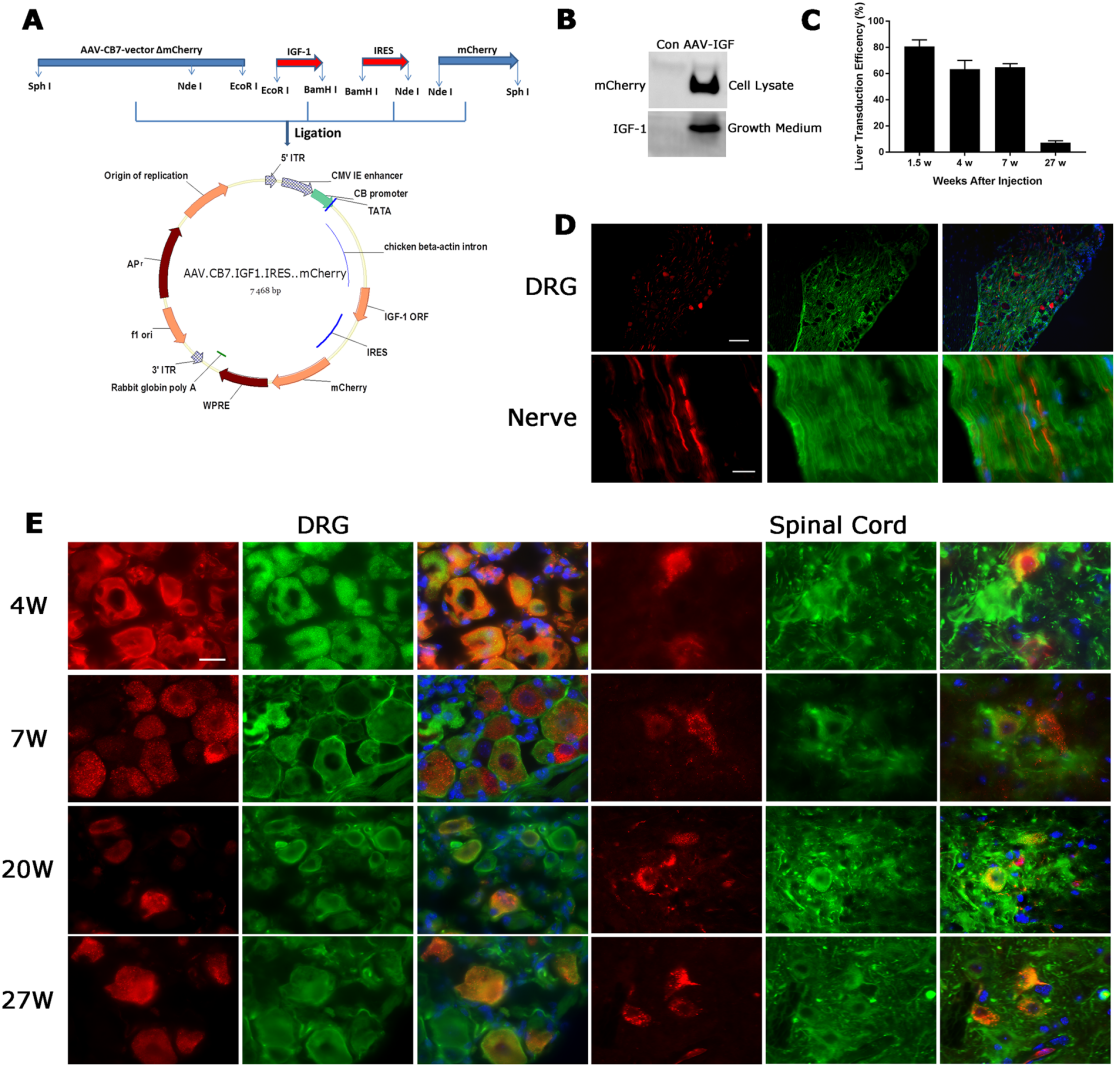
AAV-IGF-1 vector construction and the expression and distribution of IGF-1 in mice treated with AAV-IGF-1. (**A**) Cassettes of mIGF-1 cDNA, IRES, and mCherry coding region, inserted into pAAV2 vector backbone to make the new vector pAAV-IGF-1. (**B**) Western blotting showing the expression of mCherry and IGF-1 in AAV-IGF-1 transfected HEK293T cells and growth media. (**C**) The transduction efficiency of AAV-IGF-1 in mouse liver over time was evaluated by the percentage of GFP^+^ cells. (**D**) Representative immunofluorescence images showing IGF-1-mCherry (red) does not express in Schwann cells (green, anti-Sox10 antibody). Scale bars: top DRG sections = 100 µm, bottom nerve sections = 20 µm. (**E**) Representative immunofluorescence images showing the expression of IGF-1-mCherry (red) in DRG neurons (green; anti-βIII tubulin antibody) and spinal cord motor neurons (green; SMI32 antibody) of AAV-IGF-1 treated mice sacrificed at 4-, 7-, 20-, and 27-weeks post-injection. Scale bars = 20 µm.

To visualize IGF-1 expression cells, we prepared the construct carrying both mouse IGF-1 and reporter mCherry gene under the control of a universal promoter, cytomegalovirus enhancer/β-actin (CB7) promoter. The sequence of internal ribosomal entry site (IRES) was introduced into downstream of the mIGF-1 open reading frame (ORF) but upstream of the mCherry coding region, the IRES element recruits ribosome and allows second ORF of mCherry translation initiation in the middle of mRNA (Fig. 1A). The effectiveness of IGF-1 gene delivery was validated in HEK293T cells and mouse primary DRG neurons by detecting cellular mCherry expression using fluorescent microscopy (data not shown) and western blots (Fig. 1B). We then analyzed the *in-vivo* expression and distribution of AAV9-IGF-1 vectors in C57BL/6 J and B7-2^−/−^ mice at weeks 1.5, 4, 7, 20, and 27 after a single intravenous (IV) injection of AAV9-mCherry (1 × 10^13^−5 × 10^14^ vg/kg). The reporter gene (mCherry) expression can be detected in muscle, liver, heart and DRG neurons (data not shown) at 1.5 weeks after vector injection at all doses. However, mCherry expression was greatly decreased in liver (Fig. 1C) and heart, and was non-detectable in muscle at week 27 post injection (data not shown). In the nervous system, we found that mCherry expression do not co-localize with astrocytes marker GFAP (data not shown) or pan-Schwann cells marker Sox-10 (Fig. 1D). But mCherry expression was detected within spinal motor neurons and DRG neurons as early as 4 weeks post virus administration with dose from 1 × 10^14^−5 × 10^14^ vg/kg, and the mCherry signal persisted up to 27 weeks in neuronal cells after injection (Fig. 1E). Based on these data, as well as the published IV dose used for AAV9 systemic delivery to neurons^22^, we selected 2 × 10^14^ vg/kg as our experimental dose and used it in all subsequent studies.

### Systemic IGF-1 gene delivery at presymptomatic stage ameliorates peripheral nerve injury in an animal model of CIDP

The risk of relapse in CIDP patients provides the rationale for treating the disease at the presymptomatic stage. As previously described, B7-2^−/−^ mice begin to exhibit mild hind leg weakness at 20 weeks of age^13^. We first investigated the efficacy of IGF-1 gene therapy in B7-2^−/−^ mice at the presymptomatic stage. AAV9-IGF-1, AAV-mCherry (vector), or PBS vehicle was administered to presymptomatic female animals (18 weeks old). Behavioral tests (rotarod and neuromuscular severity scores (NMSS) were used in conjunction with electrophysiology (nerve conduction test) to monitor the motor function of IGF-1 treated- or control treated-animals. NMSS is based on the severity of weakness and uses a 6-point scale, as described^23^: 0 = normal strength, 1 = tail weakness only, 2 = mild/moderate fore or hind limb weakness, 3 = severe fore or hind limb weakness, 4 = mild-to-moderate fore and hind limb weakness and 5 = severe fore and hind limb weakness.

**Figure 2:**
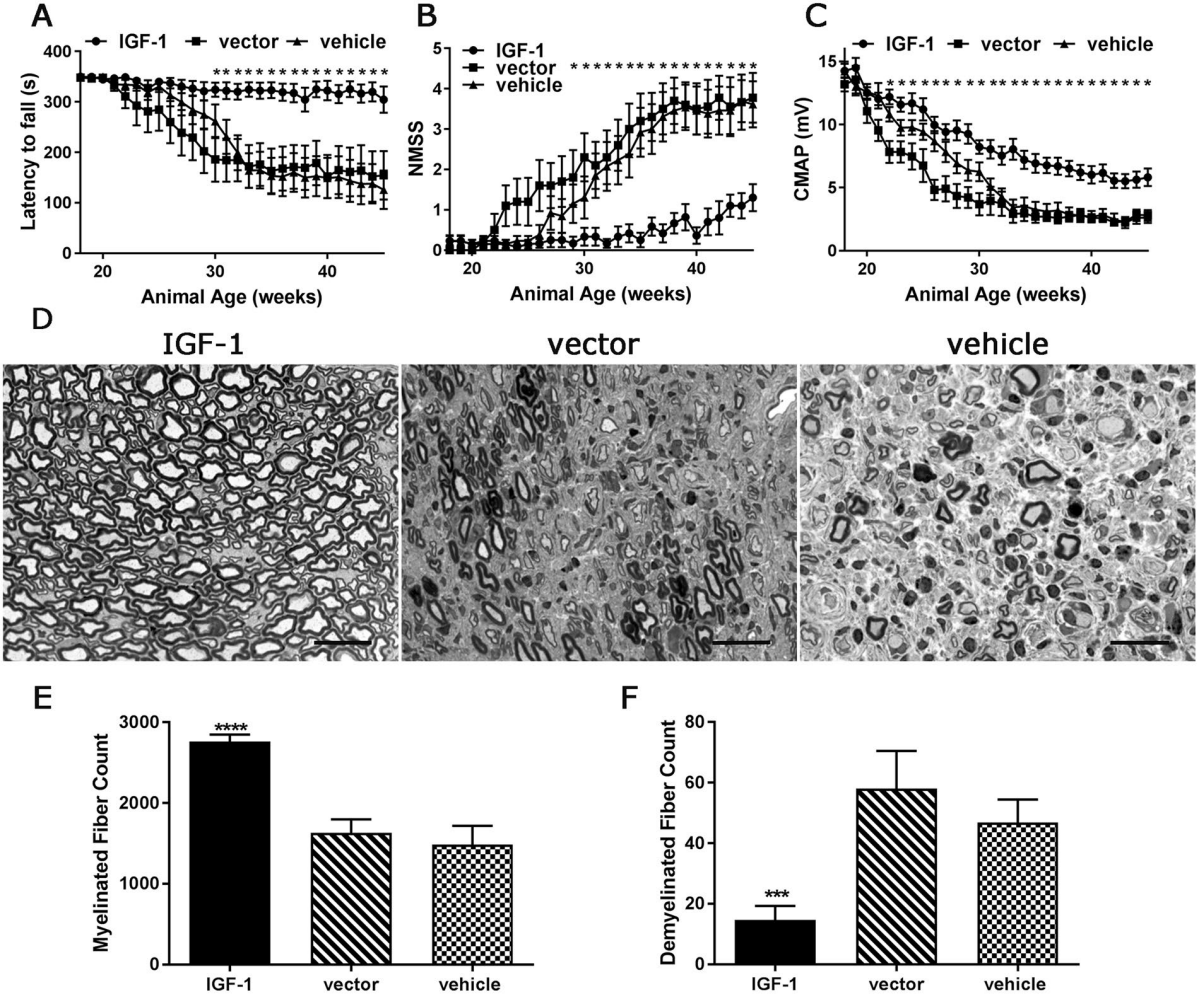
AAV-IGF-1 provides neuroprotection in female B7-2^−/−^ NOD mice at presymptomatic stage. AAV-IGF-1 or control treatments were administered to presymptomatic B7-2-null NOD mice (18 weeks old). (**A**) rotarod, (**B**) NMSS, and (**C**) electrophysiology analyses demonstrate that the neuroprotective effects were found in animals receiving AAV-IGF-1. (**D**) Representative micrographs showing many myelinated nerve fibers in sciatic nerve of AAV-IGF-1 treated mice while few myelinated axons in animals with vector or vehicle treatments. Morphometric analysis showing significant more myelinated nerve fibers (**E**) and less demyelination (**F**) in sciatic nerves from animals receiving AAV-IGF-1. Error bars represent s.e.m. Scale bars = 20 μm. *p < 0.05, ***p = 0.001, ****p = 0.0001.

Prior to the treatment, the baseline rotarod performance in all three groups was similar (mean latency to fall ~350 s). However, progressive deterioration in rotarod performance was observed in animals treated with vector or vehicle control. IGF-1 treated-mice only showed a mild decline in rotarod performance. The difference in rotarod performance between IGF-1 treated- and control treated-mice reached statistical significance by 30 weeks’ of age. The mean latency of IGF-1 treated animals was 315–325 seconds compared to 180–190 seconds in the control groups (Fig. 2A).

We further compared the motor function of IGF-1 treated-animals to that of control animals using NMSS. Previous studies in B7-2^−/−^ mice have used mean NMSS 1 as disease onset^24^. As expected in this chronically progressive SAPP model, we found that control vector-treated group demonstrated motor signs as early as 23 weeks of age, while vehicle-treated animals depicted motor deficits at age 29 weeks, and the NMSS of these control groups gradually increased over time. In IGF-1 treated animals, the NMSS were statistically significantly lower compared to control groups starting at 29 weeks of age (mean NMSS of IGF-1 treated group was 0.1 compared to vehicle-treated group of 1.2, and reporter-gene vector treated group of 1.8). Notably, using the NMSS score of =/>1 as the onset of SAPP, the neuropathic disease onset in IGF-1 treated-mice was delayed to 43 weeks of age (Fig. 2B). IGF-1 treatment also significantly improved the motor neurophysiological parameter, compound muscle action potential (CMAP) amplitudes (a measure of total motor axonal integrity), in B7-2^−/−^ mice. There were significantly higher CMAP responses in IGF-1 treated animals (Fig. 2C) starting at 22 weeks of age compared to control animals (mean CMAP of IGF-1 treated group was 12.20 mV compared to vehicle-treated group of 10.81 mV, and vector treated group of 7.83 mV). These behavioral and electrophysiological studies demonstrated that presymptomatic IGF-1 treatment delayed the onset of SAPP symptoms and attenuated disease severity in B7-2^−/−^ mice.

Female SAPP mice over time accumulate peripheral neuropathological changes, including intense inflammatory cell infiltration, demyelination, and axonal degeneration, as described previously^25^. Examination of 1-um epon sections showed that sciatic nerve samples collected from B7-2^−/−^ control groups (45 weeks old) exhibited significant inflammation, demyelinating changes, and loss of myelinated nerve fibers. In contrast, IGF-1 treated group showed a substantial reduction in inflammation, demyelination, and loss of myelinated nerve fibers (Fig. 2D). Morphometric analysis of sciatic nerve sections showed that IGF-1 treated animals had significantly more myelinated nerve fibers, but remarkably fewer demyelinated fibers than the two control groups (Fig. 2E). Overall, these studies indicate that IGF-1 treatment at presymptomatic stage protects the peripheral nerves from injury in B7-2^−/−^ mice, and support the notion that IGF-1 therapy can be used in CIDP patients as a maintenance treatment to prevent relapses.

### IGF-1 treatment at presymptomatic stage of SAPP significantly suppressed endoneurial inflammation

**Figure 3:**
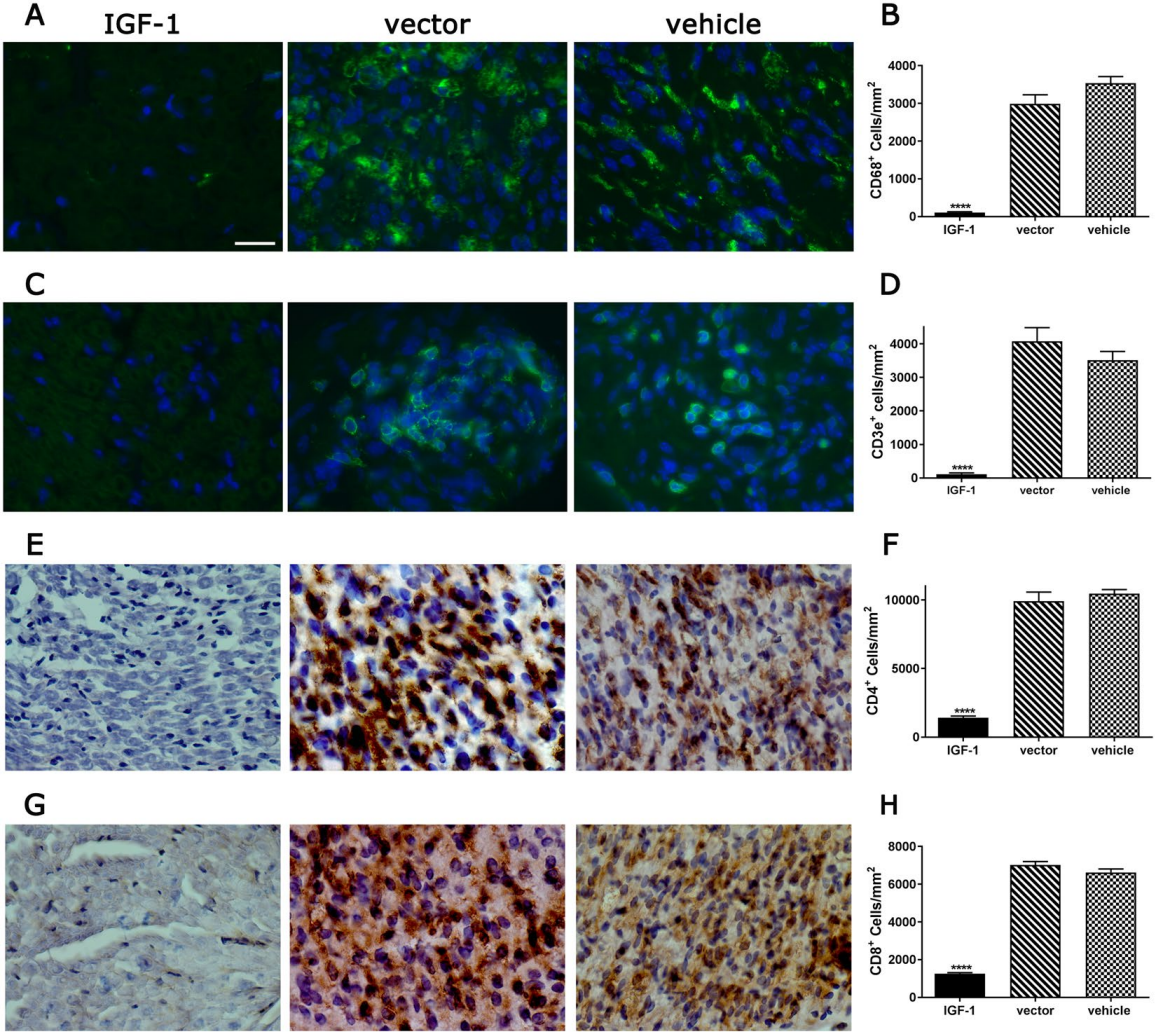
AAV-IGF-1 suppresses endoneurial inflammation in female B7-2^−/−^ NOD mice. (**A–H**) Immunohistochemical micrographs and quantitative immunohistochemistry analysis showing significant reduction of endoneurial macrophages (CD68; **A** & **B**), CD3^+^ T cells (**C** & **D**), CD4^+^ T cells (**E** & **F**), and CD8^+^ T cells (**G** & **H**) in sciatic nerves of IGF-1-treated mice compared to that of control mice. Error bars represent s.e.m. (n ≥ 3) per group. Scale bars = 20 μm. ****p < 0.0001.

Development of SAPP in B7-2 ^−/−^ mice is associated with endoneurial CD4^+^ T-cell inflammation and an increase in CD4^+^ and CD8^+^ T cells are found in this compartment in female B7-2^−/−^ mice^26^. Whether IGF-1 treatment in B7-2^−/−^ mice alters endoneurial inflammation was examined by immunocytochemistry using macrophage and T lymphocyte markers. We found that there was a significant reduction in the activated macrophage/microglia lineage cells (CD68^+^) in IGF-1 treated nerves compared to controls (Fig. 3A,B). Moreover, we found that the infiltration of total T lymphocytes (CD3e^+^, Fig. 3C,D), as well as CD4^+^ (Fig. 3E,F) and CD8^+^ (Fig. 3G,H) T cells was remarkably reduced in the sciatic nerves of IGF-1 treated group compared to controls.

**Figure 4:**
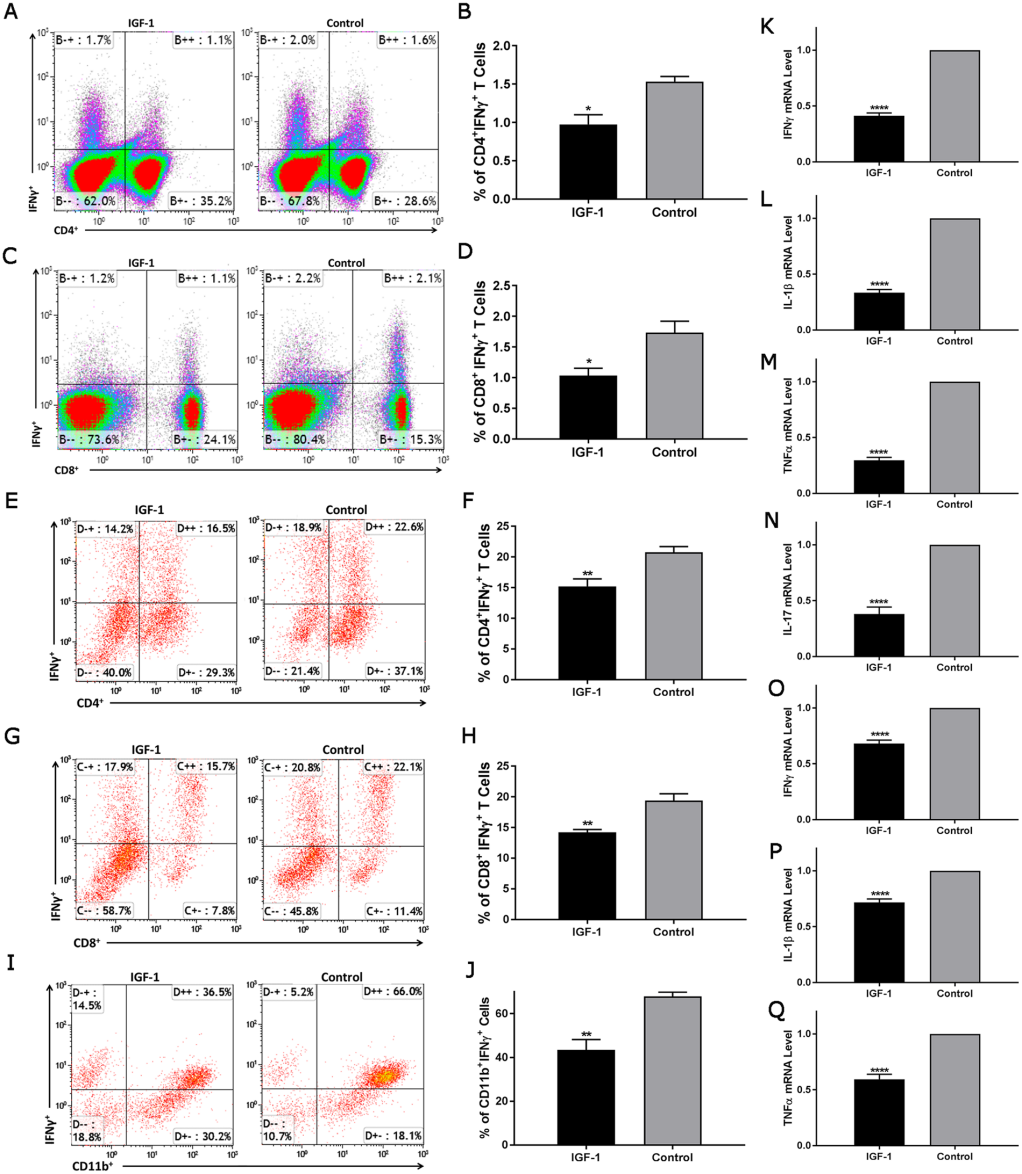
AAV-IGF-1 inhibits the production of proinflammatory cytokines. (**A–D**) Significant reductions in CD4^+^/IFNγ ^+^ (**A** and **B**) and CD8^+^/IFNγ ^+^ cells (**C** and **D**) were found in the spleens of AAV-IGF-1-treated animals by flow cytometry. (**E–J**) In addition, our *in-vitro* studies demonstrated that IGF-1 treatment significantly decreases the frequency of CD4^+^/IFNγ ^+^ (**E** and **F**) and CD8^+^/IFNγ ^+^ cells (**G** and **H**) in splenocyte culture, and reduces the frequency of CD11b^+^/IFNγ ^+^ cells in LPS stimulated RAW 264.7 cell culture (**I** and **J**). (**K–Q**) IGF-1-treatment suppresses the mRNA expression levels of pro-inflammatory cytokines (IFNγ, IL-1β, TNFα, and IL-17) in cultured splenocytes (**K–N**) or RAW 264.7 cells (**O–Q**). Error bars = s.e.m, n = 3–5 per group. *p < 0.05, **p < 0.01, ****p = 0.0001.

Previous work indicates that IFNγ is essential for the development of neuropathy in NOD B7-2^−/−^ mice, as B7-2^−/−^ mice deficient in IFNγ are completely protected from SAPP phenotype^14^. To study the mechanisms of IGF-1 mediated anti-inflammatory activity, we evaluated the IFNγ expression after IGF-1 treatment. We compared the frequencies of IFNγ producing T cells in splenocytes of IGF-1 treated and control animals. The flow cytometric analysis showed that there was a significant reduction of CD4^+^/ IFNγ^+^ and CD8^+^/ IFNγ^+^ lymphocytes (Fig. 4A–D) in IGF-1 treated animals compared to controls. To further characterize IGF-1-mediated inhibition of IFNγ expression, *ex vivo* studies were performed. Splenocytes isolated from treatment naïve B7-2^−/−^ mice were cultured and the frequency of IFNγ producing T cells was measured with and without IGF-1 treatment. Our data showed both CD4^+^/ IFNγ ^+^ and CD8^+^/ IFNγ^+^ lymphocytes were significantly decreased in IGF-1 treated splenocytes compared to controls (Fig. 4E–H). In addition to T-lymphocytes, we also tested IGF-1 inhibition of IFNγ production in the RAW 264.7, a mouse macrophage/monocyte, cell line. RAW 264.7 cells were treated with IGF-1 and the frequencies of IFNγ producing cells were measured by flow cytometry. As shown in Fig. 4I,J, there was a significant reduction of CD11b^+^/IFNγ^+^ cells in IGF-1 treated group compared to untreated control cells.

In addition to IFNγ, proinflammatory cytokines IL-1β, IL-17, and TNFα are highly expressed in activated lymphocytes and/or macrophages. To further explore the effects of IGF-1 on inflammation, splenocytes isolated from treatment naïve female B7-2^−/−^ mice were incubated with PMA/ionomycin in the presence or absence of IGF-1 for 24 hours, and these proinflammatory cytokine levels were evaluated using RT-PCR. As shown in Fig. 4K–N, IFNγ, IL-1β, IL-17, and TNFα mRNA were significantly down-regulated in IGF-1 treated cells compared to untreated controls. In corresponding *in vitro* studies on macrophage/microglia cell line, we found that these proinflammatory cytokines were down-regulated in IGF-1 treated, LPS-activated RAW264.7 cells (Fig. 4O–Q) compared to LPS-activated RAW264.7 cells without IGF-1 treatment. Viability data by flow cytometry analysis show that IGF-1 does not increase splenocyte (Supplementary Fig. S2A,B) or RAW 264.7 cell death (Supplementary Fig. S2C,D). Our findings support the notion that IGF-1 mediated protection in SAPP model might partly be through its inhibition of proinflammatory cytokine expression.

As a pleiotropic growth factor with mitogenic properties, IGF-1 has been shown to stimulate proliferation of both human and mouse regulatory T (Treg) cells *ex vivo*^27^. Tregs play an important role in inhibition of immunopathology and autoimmune disease^28^. Animal studies indicate that there are lower frequencies of Tregs in female B7-2^−/−^ mice compared to male B7-2^−/−^ and wild type NOD mice^29^. To characterize the possible signal transduction pathway of IGF-1 mediated immunomodulatory effects, we evaluated IGF-1 activity on Tregs in SAPP animals. Flow cytometry analysis was performed using splenocytes isolated from female B7-2^−/−^ mice at the termination of treatment studies. Our data showed that there is no difference in the frequency of CD4^+^/FoxP3^+^ cells between IGF-1 injected animals and controls (Supplementary Fig. S3), suggesting that IGF-1-mediated anti-inflammatory effects in SAPP model are not through stimulation and proliferation of Tregs.

### IGF-1 provides neuroprotection at symptomatic stage of SAPP

**Figure 5:**
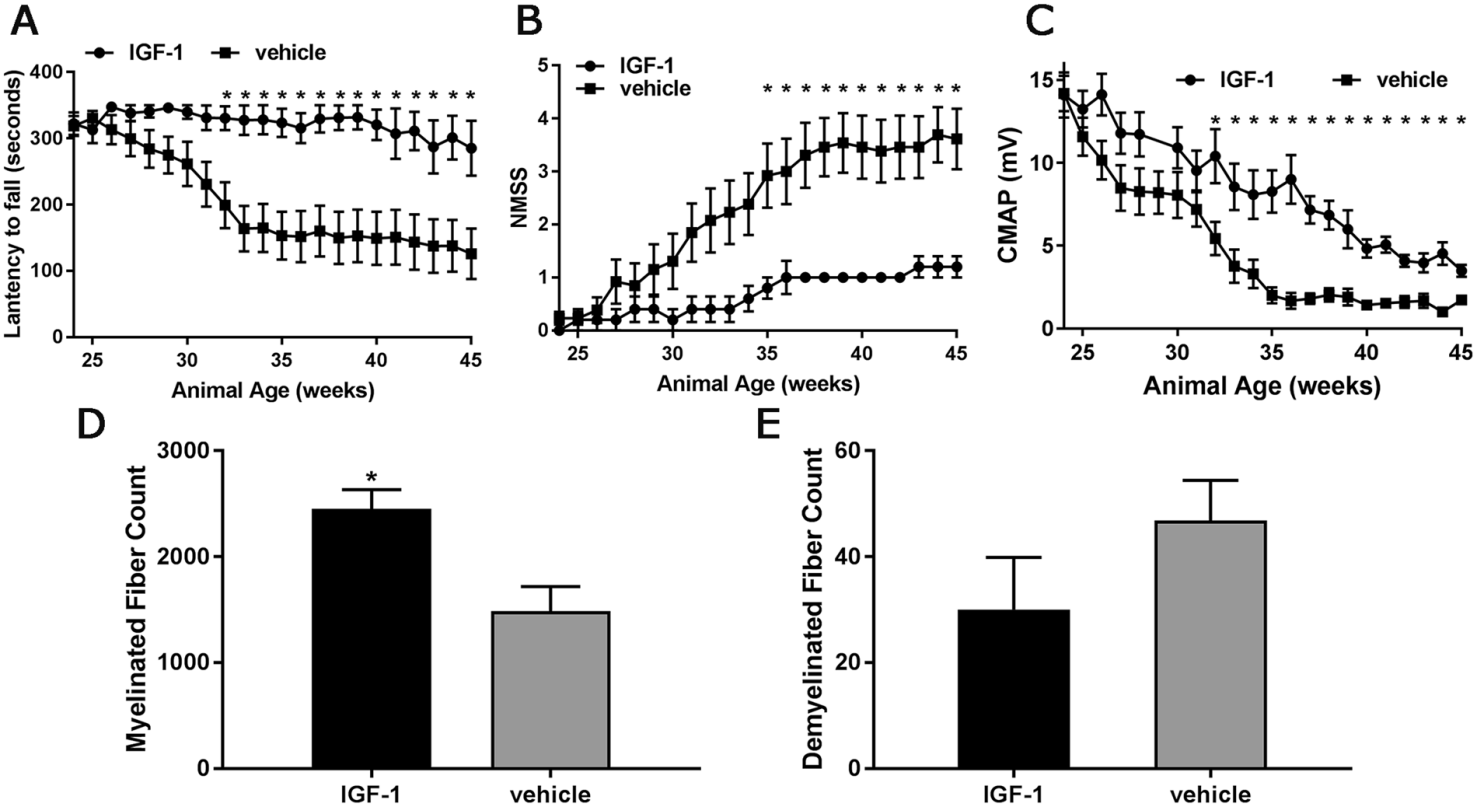
AAV-IGF-1 attenuates peripheral nerve injury in B7-2^−/−^ NOD mice at symptomatic stage. 24 weeks old female B7-2-null NOD mice received either AAV9-IGF-1 (2 × 10^14^ vg/kg) or vehicle treatment. (**A–C**) Behavioral, Rotarod (**A**); NMSS (**B**); electrophysiological (**C**), tests demonstrated that AAV-IGF-1 significantly ameliorates the clinical severity of peripheral neuropathy in those mice. (**D** and **E**) Morphometry analysis showing IGF-1 treatment increases the number of myelinated nerve fiber (**D**) while reducing demyelination (**E**). Error bars represent s.e.m. *p < 0.05.

Our results demonstrated the beneficial effects of IGF-1 treatment at presymptomatic stage in B7-2^−/−^ mice. Next, we examined the therapeutic potential of IGF-1 gene therapy in SAPP mice at the symptomatic stage. AAV9-IGF-1 (2 × 10^14^ vg/kg) or vehicle treatment was given to 24 weeks old B7-2^−/−^ mice. The behavioral performance of all animals was evaluated by rotarod and NMSS at weekly intervals until age 45 weeks. Morphometry and immunocytochemistry were performed on tissues at the end of this period. The rotarod and NMSS performance of IGF-1 treated animals were significantly better than control groups. The difference in rotarod performance reached statistical significance around age 32 weeks (mean latency to fall 330 seconds in IGF-1 treated group compared to 199 seconds in controls, Fig. 5A), and significant difference in NMSS was reached at age 35 weeks (mean NMSS 0.8 in IGF-1 treated group compared to 2.92 in control group, Fig. 5B). In addition, the CMAP amplitudes of IGF-1 treated animals were significantly higher than controls at age 32 weeks (mean CMAP of 10.41 mV in IGF-1 treated animal compared to 5.43 mV in control group, Fig. 5C). The morphometric analysis showed that IGF-1 treated animals had significantly more myelinated axons than the control mice (Fig. 5D) and the nerve fiber demyelination was reduced in IGF-1 treated group compared to control group, however, there was a trend but this difference did not reach statistical significance (Fig. 5E).

## Discussion

In this study, we report the therapeutic potential of IGF-1 in an animal model of CIDP. Systemic delivery of IGF-1 gene via recombinant adenovirus successfully postpones disease onset and reduces the severity of SAPP after disease onset in NOD B7-2^−/−^ mice. AAV-IGF-1 treatment improves motor function decline, which is associated with reduced demyelination and axonal loss. Our data indicated that a single dose of AAV vector encoding IGF-1 administered through intravenous route resulted in widespread IGF-1 expression within PNS, and the overexpression of IGF-1 in motor and sensory neurons could be detected up to 6 months. In addition, IGF-1 treatment significantly decreased the endoneurial inflammatory cell infiltration and inhibited the production of proinflammatory cytokines including IFNγ. Notably, IGF-1 gene therapy ameliorated peripheral nerve injury at both presymptomatic and symptomatic stages of SAPP, these findings are relevant to both chronic-progressive and relapsing-remitting forms of CIDP.

IGF-1 is a pleiotropic protein that exerts potent effects on neuronal survival and neurite outgrowth. In the past three decades, the efficacy of IGF-1 had been reported in animal models of neurodegenerative disease including amyotrophic lateral sclerosis (ALS), Alzheimer’s disease, and various peripheral neuropathies^30^. IGF-1 is tightly regulated by a family of at least seven high-affinity IGF-binding proteins (IGFBPs)^31^, that control the bioavailability of IGF-1 and its interaction with the IGF-1 receptor. In serum, the majority of IGF-1 generated from liver exists as a 150KDa ternary with IGFBP3 and acid labile subunit, which makes it difficult for IGF-1 to cross the capillary barrier^32^. This impermeability might have contributed to the failure of phase III clinical trial of recombinant human IGF-1 in ALS patients^33^. Studies suggest that lack of sufficient delivery of IGF-1 to target tissues make IGF-1 therapy difficult to translate from animal models to human clinical studies^34,35^.

In the present study, we report an effective neuronal delivery of IGF-1 gene using AAV9 as a vector in SAPP model. We found that as early as day 10 post virus injection, a distinct expression of the IGF-1 reporter gene, mCherry, in liver, heart, kidney and muscle as well as DRG neuronal cells. Four weeks after injection, mCherry expression was detected in spinal cord and brain neuronal populations. Our results also demonstrated that systemic delivery of rAAV9/IGF-1 induced long-term expression of IGF-1 in mice, it was detectable in the nervous systems for up to 27 weeks, but not other organs, such as liver, which is consistent with previous reports^36^. These data also suggest that the neuroprotective and/or immunomodulatory effects of IGF-1 on SAPP pathogenesis are partially auto/paracrine in nature due to local IGF-1 production in the PNS. It has been shown that autocrine/paracrine effects of IGF-1 play more important role in providing neuroprotection compared to serum IGF-1^37^. In the current study, we focused on how to enhance the local expression of IGF-1 in the PNS. The selective transfer of IGF-1 gene to nervous system not only overcomes the problem of exogenous IGF-1 protein passing through the blood-nerve and blood-brain barriers (BNB and BBB) but also bypasses the need for repeated dosing. AAV serotypes are widely used as a vehicle for therapeutic gene delivery to the nervous system because of their ability to transduce postmitotic neuronal cells and allow stable exogenous gene expression in the nervous system^38^. Among all the serotypes of AAVs, AAV9 readily crosses BNB and BBB in both neonatal and adult mice^36,39^, which expanded the applications of intravenous AAV administration in PNS and CNS gene therapy. Currently, AAV9 has been used as gene delivery vector in two phase I clinical trials for neurological disorders (Spinal Muscular Atrophy type 1, ClinicalTrials.gov Identifier: NCT02122952, and Pompe, glycogen storage disorder, ClinicalTrials.gov Identifier: NCT02240407).

Our study showed that there was a remarkable reduction of inflammatory cell infiltration in peripheral nerves. Further, we found that IGF-1 treatment significantly decreased the number of IFNγ positive CD4^+^ and CD8^+^ cells. These studies suggest that IGF-1 mediated beneficial effects in this animal model are partly attributable to its anti-inflammatory activity. Similar to CIDP, SAPP is an autoimmune disease that affects the peripheral nerves and/or nerve roots^13,40^, with increased endoneurial inflammatory leukocytes infiltration and elevated levels of proinflammatory cytokines including IFNγ resulting in axonal demyelination and/or degeneration. In addition to being a neurotrophic factor, it has been reported that IGF-1 exhibits immunomodulatory effects^41^. IGF-1 can stimulate Th2 anti-inflammatory cytokines production/secretion (IL-10, IL-4), and suppress Th1 proinflammatory cytokine (IFNγ) production^42^, suggesting that IGF-1’s anti-inflammatory function may rely on modulation of Th1 and Th2 cytokines.

In conclusion, our studies support the potential use of IGF-1 for the treatment of autoimmune neuropathies, such as CIDP. Although the precise mechanisms for IGF-1’s beneficial effect in this preclinical model of CIDP are not completely elucidated, our data implicate that IGF-1’s therapeutic properties are partly through its anti-inflammatory activity.

## Materials and Methods

### Experimental Design

All procedures were performed under a protocol approved by the Institutional Animal Care and Use Committee at the University of Texas Health Science Center at Houston that complied with governmental guidelines. Wild type (WT) C57BL/6 J mice were obtained from the Jackson laboratory and B7-2- null breeders (B7-2^−/−^), were kindly gifted by Dr. Eroboghene E. Ubogu (University of Alabama). Female B7-2^−/−^ mice were used for gene therapy studies.

### AAV-IGF-1 and -control vectors preparation

The AAV2 plasmid pENN.AAV.CB7.CI.mCherry.WP.RBG (pAAV-mCherry), pZac2.1-luc-IRES-eGFP, and pAAV2/9 were from Penn Vector Core (University of Pennsylvania). pAAV2.CB7.IGF-1.IRES.mCherry.WP.RBG (pAAV-IGF-1) encoding IGF-1 and reporter gene mCherry was constructed as follows: murine IGF-1 cDNA were obtained from WT mouse liver using following primers, 5′-CGTGGAATTCATGTCGTCTTCACACCTC-3′ and 5′-GGGCGGATCCCTACTTGTGTTCTTCAAAT-3′ with EcoR I/BamH I site at upstream/downstream of cDNA. IRES was obtained from the vector pZac2.1-luc-IRES-GFP by PCR, using primers 5′-CTAGGGGATCCGCCCCTC-3′ and 5′-GGGGCATATGGTGGCCATATTATCATCG-3′ with BamH I and Nde I on each end, respectively. The resulting IGF-1 cDNA was digested with EcoR I and BamH I, IRES fragment was digested with BamH I and Nde I, the mCherry gene was digested from vector pAAV-mCherry with Nde I and SphI, the products were ligated into vector pAAV-mCherry EcoR I and SphI site (Fig. 1A).

AAV9 vectors encoding IGF-1-mCherry or mCherry alone (control vector) were prepared by transient transfection using pAAV-IGF-1 plasmid or pAAV-mCherry, with plasmid pAAV2/9 carrying AAV9 capsid, and adenoviral helper plasmid pHelper (Cell Biolabs) in HEK293T cells. The virus was produced and purified followed by large-scale AAV production, as described^43,44^. Briefly, one hundred 15-cm plates of HEK293T cells were grown in Dulbecco’s modified Eagle’s medium (DMEM) with 10% fetal bovine serum (FBS). The day before transfection, 1–2 × 10^7^ cells were re-plated into each 15-cm dish and transfected using polyethyleneimine (PEI; Polysciences) with PEI/plasmids ratio ~1.5: 1. The total amount of 45 μg of DNA at a ratio of 1:1:1 (pAAV-IGF-1: pHelper: pAAV2/9) were mixed with serum-free DMEM and added to each 15-cm dish. 72-120 hours post transfection, both transfected cells and medium were harvested for vector purification. Cell pellets were resuspended in gradient buffer (GB, 10 mM Tris, pH 7.6, 150 mM NaCl, 10 mM MgCl_2_), and stored at −80 °C until further processing.

Cell-associated vector was released by three sequential freeze-thaw cycles (−80 °C/37 °C). Vectors that released in culture medium were precipitated with 40% polyethylene glycol (PEG, BioUltra 8000) and 2.5 N NaCl buffer to a final PEG concentration of 8%. Vectors were harvested by centrifugation, then resuspended in GB, and combined with cell lysate. 50U/ml benzonase was added to the mixture, incubated at 37 °C for 40 minutes, and cell debris was removed by centrifugation. Vectors were purified by ultracentrifugation on an iodixanol density gradient, as described^45^. Purified vectors were desalted and concentrated with Amicon Ultra—Ultracel 30 K filter units. 0.1% pluronic-F68 (ThermoFisher) was added to prevent virus aggregation and stored at 4 °C until further use. AAV titers are given as viral genomes per ml (vg/ml), determined by real-time PCR for mCherry using specific primers.

### Viral vector administration and experimental paradigm

16 eight-to-ten weeks old C57BL/6 J female mice were used for the viral titration/dose and gene expression studies. Animals were separated into 4 groups and each group was intravenously injected with AAV9-mCherry at dose 1 × 10^13^, 5 × 10^13^, 1 × 10^14^ or 5 × 10^14^ vg/kg respectively, tissues were collected at week 1.5, 4, 7, 20 and 27 after viral injection to assess reporter gene expression and distribution.

For IGF-1 gene therapy experiments, 18 weeks (presymptomatic stage; n = 13) and 24 weeks (symptomatic stage; n = 5) old female B7-2^−/−^ mice were intravenously injected AAV9-IGF-1 (2 × 10^14^ vg/kg). Controls included age and sex matched B7-2^−/−^ mice injected with the same dose of AAV9-mCherry vector (age 18 weeks, n = 12) or vehicle/PBS (n = 19 at age 18 weeks and n = 5 at age 24 weeks). Behavior and nerve conduction testing (see below) were performed at baseline prior to injections and weekly thereafter. The studies were terminated at 27 weeks post-injection (presymptomatic stage) or 21 weeks post-injection (symptomatic stage) and animals were perfused and tissues were harvested for morphological and immunocytochemical studies.

### Behavioral Testing

Rotarod (Med Associates) testing and NMSS were performed weekly on all animal groups to assess coordination and motor strength, respectively. Each session consisted of three trials on the accelerating rotarod. The latency to fall from the rod was measured for each mouse. NMSS is based on the severity of weakness and uses a 6-point scale, as described^23^: 0 = normal strength, 1 = tail weakness only, 2 = mild/moderate fore or hind limb weakness, 3 = severe fore or hind limb weakness, 4 = mild-to-moderate fore and hind limb weakness and 5 = severe fore and hind limb weakness.

### Electrophysiology

Sciatic motor nerve conductions were performed bilaterally at baseline and at weekly intervals after viral/vehicle injections in all treatment groups, as described^46^.

### Immunocytochemistry (ICC)

#### Fluorescent microscopic studies

Animals were perfused with PBS and 4% paraformaldehyde and harvested tissues were fixed in 4% paraformaldehyde for ICC studies. For single and double labeling ICC studies, fixed tissues were cryoprotected and cryosectioned at 10 μm. The following primary antibodies were used to monitor reporter gene mCherry expression in specific tissues: anti-mCherry (16D7), anti-SMI32 and anti-β III tubulin (neuronal marker), anti-GFAP (GA5), anti-S100 (ab868), anti-MBP (mab386), anti-P0 and anti-Sox-10 (N-20, astrocyte and Schwann cell marker); Rat anti-CD68 (FA-11), and rat anti-CD3e (17A2) were used for detection of microglia/macrophage and lymphocytes infiltration in the peripheral nerves. Tissues were then developed with specific fluorophore labeled secondary antibodies. All sections were analyzed by fluorescence microscope.

#### Light microscopic studies

T-lymphocyte inflammation (CD4 and CD8) was examined in sciatic nerves of B7-2 ^−/−^ mice with rat anti-CD4 (GK1.5) and anti-CD8a (53–6.7) antibodies and developed sequentially with biotinylated goat anti-rat secondary antibody, VECTASTAIN Elite ABC HRP Kit, and SG peroxidase substrate. Light microscopy was performed, as described^13^.

### Morphometry

Sciatic nerves were harvested from B7-2^−/−^ mice and fixed in 3% glutaraldehyde, embedded in epon, and 1 μm cross sections were stained with toluidine blue, as described^47^. All myelinated axons in a single whole cross-section of the nerve were counted at light level (40×) by using a motorized stage and stereotactic imaging software Axiovision (Zeiss), as described^46^. Demyelination was quantified by counting completely demyelinated axons and thinly myelinated fibers (representing demyelinated and remyelinated nerve fibers) in a single whole cross-section of the nerve.

### Splenocytes isolation and flow cytometry analysis

Spleens were isolated from 45 weeks old animals (n = 3 from each treatment group) and RBCs were lysed with Ammonium-Chloride-Potassium (ACK) buffer. Single-cell suspensions were prepared in RPMI 1640 containing 10% FBS. Splenocytes were stimulated with PMA (5 ng/ml)/ionomycin (0.5 μg/ml) and protein transport inhibitor GolgiStop (2 μM) for 4 hours. The stimulated splenocytes were first stained with anti-CD4-PerCP 710 (RM4–5) and anti-CD8-FITC (53–6.7). After permeabilization, these cells were stained with anti-IFNɣ-PE (XMG1.2). For *ex-vivo* experiments, splenocytes isolated from 25–35 weeks old treatment naïve animals were first stimulated with CD3/CD28, with or without 400 ng/ml of IGF-1 (R&D Systems) for 4 days, then incubated with PMA/ionomycin/GolgiStop for 4 hours. IGF-1 inhibition of IFNɣ production was also examined in mouse monocyte/macrophage cell line RAW 264.7 stimulated with 1 μg/ml lipopolysaccharides (LPS, Sigma-Aldrich cat# L8274), in the presence or absence of 100 ng/ml mIGF-1 overnight, treated with GolgiStop for additional 4 hours. Splenocytes were stained with CD4/CD8/IFNɣ antibodies, RAW264.7 cells were stained with anti-CD11b-APC (M1/70) and anti-IFNɣ-PE (ThermoFisher). The cells were then analyzed with flow cytometer (Beckman-Coulter Gallios Flow Cytometer) using Kaluza software (Beckman-Coulter).

### Cell cultures

Primary DRG neuron cell cultures were performed, as described^48^, to validate viral vector infection and recombinant target proteins expression in neuronal cells. Viral vectors were mixed with DRG cultures and plated in 6-well dishes. 7 days after infection, DRG cultures were examined for mCherry expression by fluorescent microscopy.

### mRNA expression of proinflammatory cytokines

Splenocytes isolated from treatment naïve B7-2 ^−/−^ mice were stimulated with PMA/ionomycin in the presence or absence of mIGF-1 for 24 hours. Similarly, RAW 264.7 cells were stimulated with LPS with or without IGF-1 for 24 hours. Total RNA was extracted from splenocytes and RAW 264.7 cells using Trizol reagent, and cDNA was generated using cDNA Synthesis Kit, and Real-time PCR was performed for the detection of cytokines using specific primers listed in Table 1.

**Table 1:** Primers used for Real-Time PCR experiments.

Gene		Primer Sequence (5′−3′)	Accession number
IL-1 β	F	CCCAAGCAATACCCAAAGAA	NM_008361
	R	GCTTGTGCTCTGCTTGTGAG	
IL-17	F	TTCAGGGTCGAGAAGATGCT	NM_010552
	R	AAACGTGGGGGTTTCTTAGG	
IFNγ	F	GCGTCATTGAATCACACCTG	NM_008337.4
	R	TGAGCTCATTGAATGCTTGG	
TNFα	F	TAGCCAGGAGGGAGAACAGA	M38296.1
	R	TTTTCTGGAGGGAGATGTGG	
18 s	F	ACCGCAGCTAGGAATAATGGA	NG_054875.1
	R	GCCTCAGTTCCGAAAACCA	

IL-1β: interleukin 1 beta; IL-17: interleukin 17; IFNγ: interferon gamma; TNFα: tumor necrosis factor alpha; 18 S: 18 S ribosomal RNA.

### Western Blotting

Plasmids encoding IGF-1 and mCherry were delivered into HEK293T cells. 48 hours post transfection, cells lysate and culture medium were collected for protein assay. Heat-denatured samples were separated on a 12% SDS Gel and transferred to a PVDF membrane and probed with goat anti-mouse IGF-1 (R&D Systems) or rabbit anti-mCherry antibodies. PVDF membranes were developed with appropriate HRP-conjugated secondary antibodies (Jackson ImmunoResearch) and a chemiluminescent reagent (SuperSignal; ThermoFisher). Signal was detected and quantified with ChemiDoc XRS system (BioRad).

### Statistics

Data are reported as mean±s.e.m. Differences among groups were examined by Student’s t-tests, or Tukey’s post hoc test after one-way or two-way ANOVA analysis. *P* < 0.05 was considered statistically significant.

## Electronic supplementary material

Dataset 1
