# Supplementary material for: Systemic IGF-1 gene delivery by rAAV9 improves spontaneous autoimmune peripheral polyneuropathy (SAPP)
Source: Sci Rep. 2018 Apr 3;8:5408. doi: 10.1038/s41598-018-23607-9 (PMC5883061; doi:10.1038/s41598-018-23607-9)

**SUPPLEMENTARY INFORMATION**

**Systemic IGF-1 gene delivery by rAAV9 improves spontaneous autoimmune peripheral polyneuropathy (SAPP)**

Tong Gao, Nataliia Bogdanova, Sameera Ghauri, Gang Zhang, Jianxin Lin and Kazim Sheikh*

Department of Neurology, McGovern Medical School at The University of Texas Health Science Center at Houston, Houston, TX, 77030 USA

**Correspondence author E-mail**: [kazim.sheikh@uth.tmc.edu](mailto:kazim.sheikh@uth.tmc.edu)

**Supplementary Figures**

**Supplementary Figure S1.** Full blots of cropped blot shown in Fig. 1B. (A) Full blot of the expression of mCherry in AAV-IGF-1 transfected HEK293T cell lysate. (B) Full blot of the expression of IGF-1 in transfected HEK293T cell growth media.


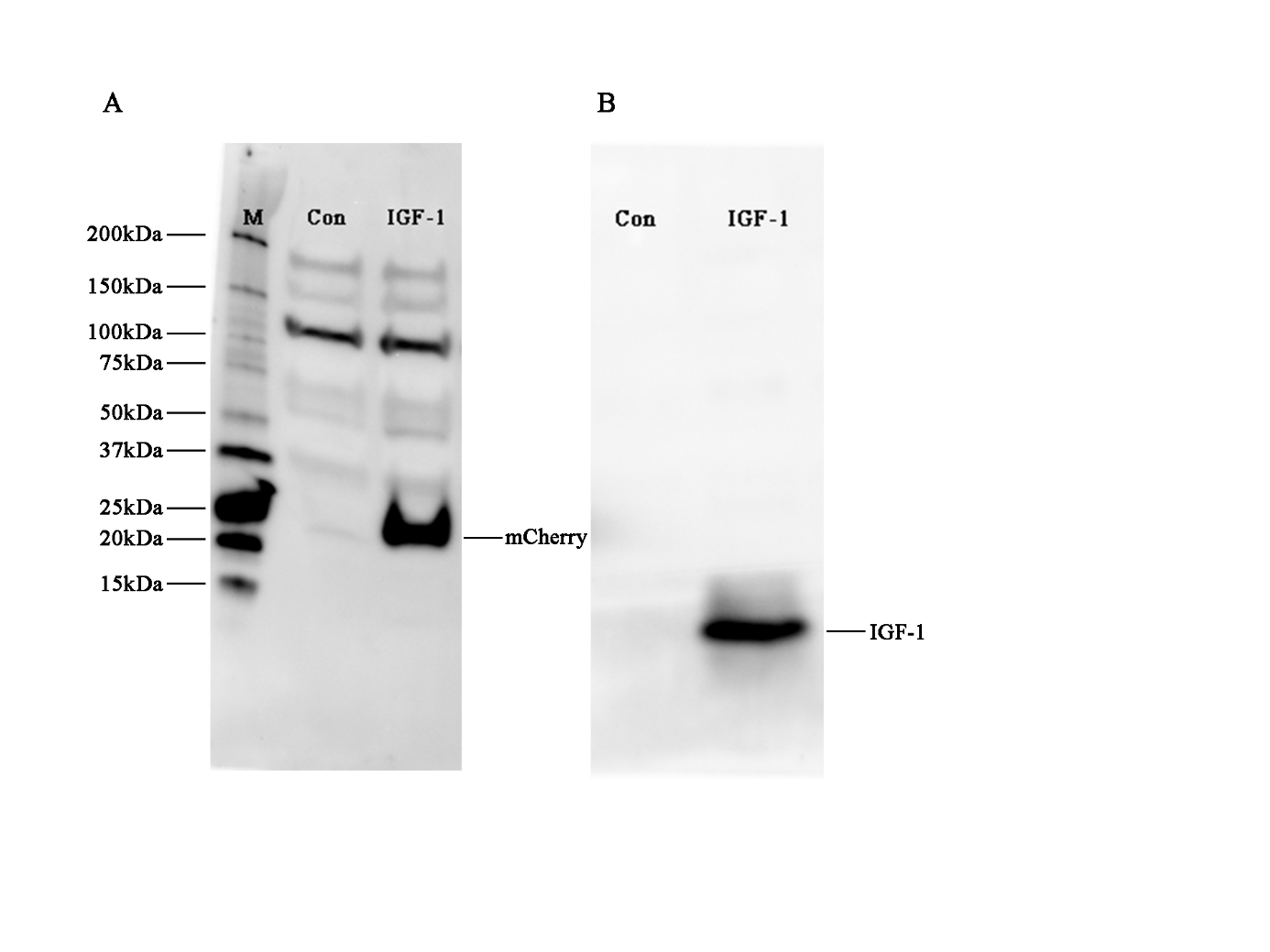


**Supplementary Figure S2.** IGF-1 treatment does not increase T-cell death in vitro. (A) Representative plot of splenocytes viability after stimulation with PMA/ionomycin in the presence (IGF-1) or absence (control) of IGF-1 and (B) quantification of viability. (C) Representative plot of RAW264.7 cell viability after LPS stimulation overnight with or without (control) IGF-1 treatment and (D) quantification of viability.


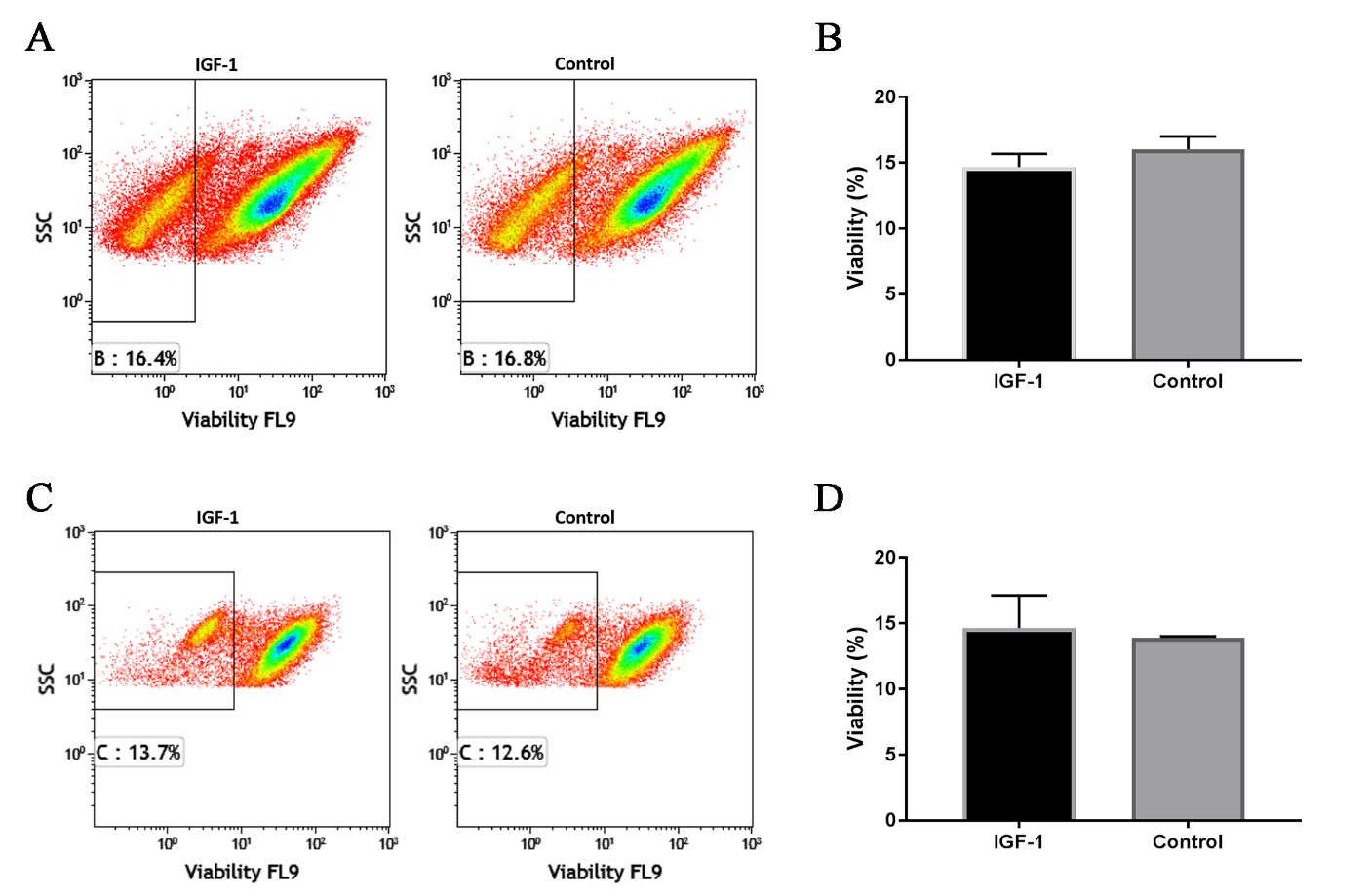


**Supplementary Figure S3.** There are no significant changes in the frequency of Tregs in SAPP animals treated with AAV-IGF-1 or AAV-mCherry. Representative flow plots (A) and quantification (B) of CD4^+^/FoxP3^+^ spleoncytes from AAV-IGF-1 (IGF-1) or AAV-mCherry (control) treated animals.


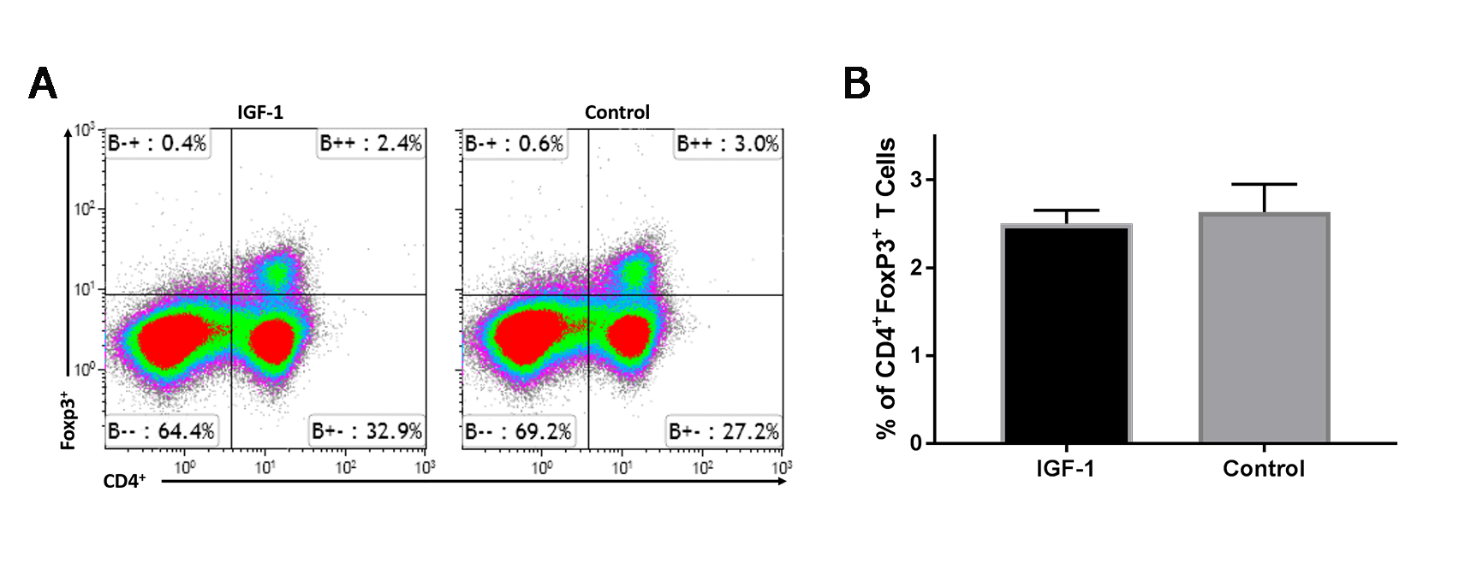

Supplement: Supplementary file 1 — Dataset 1 [file 41598_2018_23607_MOESM1_ESM.docx]
